# Adipose-derived mesenchymal stromal cell-microenvironment interaction network in metabolic syndrome: ADMSC injury response, adaptive regulation, and regenerative potential

**DOI:** 10.3389/fcell.2026.1821763

**Published:** 2026-05-07

**Authors:** Li-Yang Yuan, Qing-Yun Yuan, Ding-Wen Guo, Hui-Lan Tan, Xiao-Hui Guan

**Affiliations:** 1 The Fourth Clinical Medical College of Nanchang University, Nanchang, China; 2 The National Engineering Research Center for Bioengineering Drugs and the Technologies, Institute of Translational Medicine, Nanchang University, Nanchang, China

**Keywords:** adipose-derived mesenchymal stromal cells, exosomes, insulin resistance, metabolic syndrome, regenerative medicine, stem cell therapy

## Abstract

Metabolic syndrome (MetS) is a complex metabolic disorder characterized by insulin resistance, central obesity, dyslipidemia, hypertension, and chronic inflammation, which collectively increase the risk of type 2 diabetes mellitus, cardiovascular disease, and metabolic dysfunction-associated steatotic liver disease. Although adipose-derived mesenchymal stromal cells (ADMSCs) have shown promising therapeutic potential in preclinical studies, their efficacy is substantially influenced by the diseased local microenvironment. Accumulating evidence indicates that ADMSCs exert multi-target therapeutic effects in MetS by alleviating insulin resistance, modulating inflammation, restoring mitochondrial and redox homeostasis, regulating lipid metabolism, and protecting vascular and metabolic organ function. However, the pathological state associated with MetS, characterized by chronic inflammation, oxidative stress, and metabolic imbalance, can reciprocally impair ADMSC function and limit their therapeutic efficacy. In this review, we summarize the current mechanistic understanding of ADMSC–based therapy for MetS and highlight the bidirectional interaction between ADMSCs and the local microenvironment network, discussing how exosome-based and microenvironment-oriented strategies may support the development of more precise and effective regenerative interventions for metabolic disease.

## 1 Introduction

Mesenchymal stromal cells (MSCs) are a type of adult stromalcell with multiple biological properties. They can be isolated from multiple tissues, including bone marrow, dental pulp, placenta, umbilical cord blood, peripheral blood, and adipose tissue (Han X. et al., 2025). They exhibit self-renewal capability, *in vitro* proliferative ability, and multilineage differentiation potential. Moreover, they exhibit homing capacity toward sites of injury, inflammation, and tumors.

Adipose-derived mesenchymal stromal cells (ADMSCs) are adult stromalcells isolated from human adipose tissue, and research on these cells has progressed through several stages. In 2001, the pioneering work of the Zuk team first achieved the systematic isolation and characterization of human adipose-derived multipotent mesenchymal stem cells. More importantly, this study confirmed through *in vitro* induction experiments that ADMSCs can differentiate into adipocytes, chondrocytes, myocytes, and osteoblasts, providing early experimental evidence of their multipotent differentiation potential (Zuk et al., 2001). Based on previous research, the Zuk team further optimized the isolation procedure in 2002, systematically elucidated their immunophenotypic features and differentiation potential of ADMSCs, and innovatively proposed the concept of “adipose-derived multipotent cells,” thereby establishing a core terminology framework for subsequent research (Zuk et al., 2002).

In terms of functional characteristics, ADMSCs possess trilineage differentiation potential comparable to that of other MSCs. Their differentiation capacity can be assessed using standard histochemical methods, including Oil Red O or Nile Red staining for adipogenesis, Alizarin Red or von Kossa staining for osteogenesis, and Alcian Blue or Safranin O staining for chondrogenesis (Frenz-Wiessner et al., 2024). Under specific inductive conditions, *either in vivo or in vitro*, they can differentiate into both mesodermal and non-mesodermal lineages, including endothelial and cardiac-like cells, as well as neural and pancreatic cells (Al-Ghadban and Bunnell, 2020).

Metabolic syndrome (MetS) is a multifactorial disorder, primarily including abdominal obesity, insulin resistance, hyperglycemia, hypertension, and dyslipidemia, that markedly increases the risk of cardiovascular disease and type 2 diabetes (Alberti et al., 2009). The development of MetS is driven by an interplay of genetic predisposition, environmental factors, and lifestyle behaviors, and is characterized by the clustering of multiple cardiovascular risk factors (Obunai et al., 2007). Evidence from a meta-analysis of 37 studies demonstrates a strong association between MetS and an increased risk of cardiovascular disease (Gami et al., 2007). Among the underlying mechanisms, insulin resistance, chronic low-grade inflammation, and neurohormonal activation are considered key contributors to the progression of MetS toward overt cardiovascular disease (CVD) and type 2 diabetes mellitus (T2DM) (Fahed et al., 2022). With ongoing changes in lifestyle and dietary habits, the prevalence of MetS is on the rise, presenting a significant global public health problem (Noubiap et al., 2025). In the United States, the prevalence of MetS as defined by the Adult Treatment Panel III (ATP III) is approximately 22%, whereas the prevalence prediction is more than 30% based on the International Diabetes Federation (IDF) definition, with nearly one-third of Americans fulfilling the defining diagnosis of MetS (Moore et al., 2017).

**FIGURE 1:**
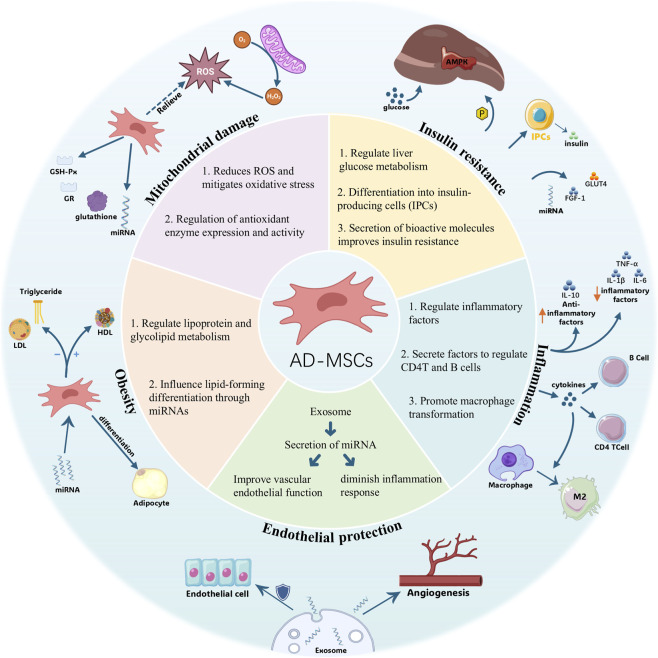
Mechanisms of adipose-derived mesenchymal stromal cells (ADMSCs) in treating metabolic syndrome (MetS). (1) Mitochondrial damage—ADMSCs reduce reactive oxygen species (ROS), alleviate oxidative stress, and regulate antioxidant enzyme expression and activity. (2) Insulin resistance—ADMSCs enhance hepatic glucose metabolism, differentiate into insulin-producing cells (IPCs), and secrete bioactive molecules that improve insulin sensitivity. (3) Lipid metabolism—ADMSCs regulate lipoprotein and glycolipid metabolism and influence adipocyte differentiation via miRNAs. (4) Inflammation—ADMSCs modulate inflammatory factors, secrete immunoregulatory molecules to influence CD4^+^ T cells and B cells, and promote macrophage polarization to the M2 phenotype. (5) Exosomes—ADMSC-derived exosomal miRNAs improve vascular endothelial function and stimulate angiogenesis.

As a systemic metabolic disorder, MetS is closely associated with adipose tissue dysfunction, chronic low-grade inflammation, oxidative stress, and persistent metabolic imbalance. ADMSCs have attracted considerable interest in the treatment of MetS because adipose tissue is abundant and can be obtained through relatively minimally invasive procedures (Fraser et al., 2006). In addition to their multilineage differentiation potential, ADMSCs exhibit immunomodulatory and paracrine activities and can be used in either autologous or allogeneic settings with relatively low immunogenicity (Mikłosz and Chabowski, 2023). These characteristics make ADMSCs a particularly promising cell source for metabolic disorders, in which adipose tissue dysfunction, chronic inflammation, and systemic metabolic imbalance play central pathogenic roles. The relevant mechanisms are illustrated in Figure 1.

Currently, the majority of treatments for MetS focus on lifestyle modifications and pharmacological interventions aimed at lowering blood glucose, improving lipid profiles, and alleviating systemic insulin resistance, whereas research on stem cell therapies remains relatively limited. Extensive preclinical studies have demonstrated that ADMSCs exert multi-targeted and multi-pathway therapeutic effects in metabolic syndrome (MetS). However, within the pathological context of metabolic disorders, the widespread pathological alterations induced by tissue damage throughout the body can counteract the therapeutic effects of ADMSCs, posing complex challenges for their clinical translation. Consequently, it is imperative to re-examine the interaction between ADMSCs and metabolic diseases from a broader and more integrated perspective.

Based on current knowledge of the mechanisms by which ADMSCs act in MetS, this review moves beyond the traditional unidirectional framework and emphasizes the bidirectional regulation of ADMSC function by the cell-microenvironment interaction network in MetS. It also explores the significant value of preserving or enhancing endogenous ADMSC function as a strategy for more precise intervention in MetS. By elucidating this cell-microenvironment interaction network, we aim to provide a theoretical basis and practical guidance for optimizing ADMSC-based therapeutic strategies.

## 2 Adipose-derived mesenchymal stromal cells

### 2.1 Key characteristics and comparisons with other MSCs

Compared with other MSC sources, such as bone marrow mesenchymal stromal cells (BMMSCs), umbilical cord mesenchymal stromal cells (UCMSCs), and dental pulp mesenchymal stromal cells (DPMSCs) (Table 1), ADMSCs can be readily isolated from adipose tissue obtained by minimally invasive procedures; standard isolation methods include enzymatic digestion and, in some settings, explant culture. They exhibit strong adipogenic potential, robust *in vitro* proliferative capacity, and relatively stable senescence characteristics. This comparison highlights the distinct value of ADMSCs in metabolism-related diseases.

**TABLE 1:** Comparison of characteristics of MSCs from different sources.

Characteristic	ADMSCs	BM-MSCs	UCMSCs	DPMSCs	Refs
Source and isolation	Subcutaneous/visceral adipose tissue; enzymatic digestion	Bone marrow cavity; aspiration + Ficoll centrifugation	Umbilical cord Wharton’s jelly/perivascular tissue; explant culture or enzymatic digestion	Pulp tissue; enzymatic digestion or transplantation method	Mushahary et al. (2018), Fong et al. (2016), Hilkens et al. (2013)
Trilineage differentiation	Strong adipogenesis; moderate osteogenesis/chondrogenesis	Strong osteogenesis/chondrogenesis; weak adipogenesis	Multipotent (transgerm layer); low adipogenesis	Strong osteogenesis; comparable adipogenesis; significant neurogenesis	Mushahary et al. (2018), Baksh et al. (2007), Labedz-Maslowska et al. (2020)
Proliferation and senescence	High proliferation; moderate senescence (donor-dependent)	Low proliferation; prone to senescence	High proliferation; low senescence (stable in passaging)	High proliferation; low senescence	Kern et al. (2006), Naeem et al. (2022), Gronthos et al. (2000)
Exosomal proteins	ATP2B1, ATP1A1 (immunomodulation)	ADAM9, ADAM10, CD81 (regeneration-related)	PAI-1, IL-6, IL-8, CD9/63/81	ALP, DSPP, DMP-1; elevated anti-inflammatory cytokines	Wang et al. (2020a), Yamada et al. (2006), Ogata et al. (2022)
Therapeutic fields	Immunomodulation; metabolic syndrome	Bone/cartilage regeneration; neural repair	Tissue repair; neurodegenerative/immune disorders	Periodontal tissue regeneration; bone tissue engineering; neural regeneration	—
General MSC-related limitations: donor variability/heterogeneity; safety verification; cancer risk; immune rejection; manufacturing standardization ADMSC-related limitations: depot-dependent heterogeneity (subcutaneous/visceral); functional variability associated with donor metabolic status; potential impairment in autologous ADMSCs from obese/diabetic donors					Ogata et al. (2022), Koste et al. (2024), Gleitz et al. (2021), Pan et al. (2023)

Abbreviations. MSCs, mesenchymal stromal cells; ADMSCs, adipose-derived mesenchymal stromal cells; BM-MSCs, bone marrow-derived mesenchymal stromal cells; UCMSCs, umbilical cord-derived mesenchymal stromal cells; DPMSCs, dental pulp-derived mesenchymal stromal cells.

This table summarizes representative comparative features of MSCs derived from different tissue sources. These characteristics may vary according to donor background, isolation protocol, and culture conditions.

ADMSCs isolated from rat adipose tissue exhibit enhanced secretion of growth factors and inflammatory cytokines, including vascular endothelial growth factor (VEGF), hepatocyte growth factor (HGF), and interleukin-6(IL-6), which may contribute to their reparative and regenerative effects (Xue et al., 2024). *Iin vitro* studies have shown that, compared with BM-MSCs, ADMSCs derived from the same individual have superior proliferation potential, suggesting that ADMSCs possess substantial developmental prospects and important potential value in the industrial application of stem cells (Peng et al., 2008). The adipogenic capacity of ADMSCs exceeded that of BMMSCs, evidenced by increased lipid vesicle formation and adipogenic gene expression (Mohamed-Ahmed et al., 2018). This property makes ADMSCs pertinent to the regulation of obesity and metabolic diseases. In metabolic diseases such as obesity and type 2 diabetes, preclinical studies suggest that ADMSCs may ameliorate obesity and dyslipidemia by regulating adipocyte production and function, thereby alleviating disease symptoms (Pham et al., 2023).

Compared with donor-matched BM-MSCs, ADMSCs may exhibit relatively favorable *in vitro* proliferative stability and lower senescence under certain culture conditions (Mohamed-Ahmed et al., 2018). A comparison between pediatric and young adult donors suggests that some basic ADMSC characteristics may be preserved across younger age groups (Abbo et al., 2017); however, this observation should not be extrapolated to typical aging; donor age and specificity, as well as abnormal pathological stress, continue to influence ADMSC phenotype and function (Delben et al., 2021).

### 2.2 Identification and influencing factors of ADMSCs

In 2001, Gronthos identified the immune surface marker profile of human ADMSCs, providing key molecular evidence for the specific identification and preliminary characterization of cells (Gronthos et al., 2001). In 2013, the International Federation for Adipose Tissue Science and Therapy (IFATS) and the International Society for Cell Therapy (ISCT) jointly established Cytotherapy, establishing a standardized reference protocol for the identification of ADMSCs, marking the entry of research in this field into a phase of standardization (Bourin et al., 2013). Current investigations lack definitive proof that any specific source of MSCs provides a more substantial therapeutic benefit regarding treatment effect. Given this, it is essential to clarify and verify the distinct properties of MSCs derived from different tissues to advance treatment methods for certain disorders.

Investigating source-dependent gene expression programs associated with MSC differentiation potential provides a molecular basis for selecting the most appropriate MSC source for specific applications (Gao et al., 2023). In addition, recent research has concentrated on surface markers of ADMSCs to optimize their identification and isolation. *In vitro* studies found that ADMSCs express specific markers, including CD29, CD34, CD44, CD166, CD73, CD90, and CD105 (Pan et al., 2019; Salamon et al., 2013; Laschke et al., 2013; He et al., 2013), which facilitate the differentiation of ADMSCs from other cell types. For example, CD34 expression gradually decreases during culture expansion (Murphy et al., 2013). Functionally, CD34^+^ ADMSCs exhibit higher expression of endothelial progenitor cell markers, whereas CD34^−^ cells show greater adipogenic differentiation potential (Suga et al., 2009).

To further elaborate on the specific expression characteristics and functional roles of the aforementioned ADMSC surface markers, Table 2 summarizes some key surface markers, providing a more detailed molecular basis for the accurate identification, separation, and functional study of ADMSCs.

**TABLE 2:** Surface markers of adipose-derived mesenchymal stromalcells.

Surface marker	Expression features	Key functions	Refs
CD29 (Integrin β1)	High (98% in P1 rat ADMSCs)	Maintains adhesion and fibroblast-like morphology	Davies et al. (2015), Sung et al. (2008)
CD34	Dynamic (positive→ negative)	Mediates lineage-specific differentiation (adipose, bone, smooth muscle)	Suga et al. (2009)
CD44 (Hyaluronic acid receptor)	Strongly positive	Regulates cell-matrix interactions; sustains high proliferation of ADMSCs	Sung et al. (2008)
CD73 (5′-Nucleotidase)	Negatively correlated with osteogenesis	Inhibits osteogenesis; generates adenosine via ATP metabolism when paired with ALP	Canepa et al. (2021)
CD90 (Thy-1)	High (73% in P1 rat ADMSCs)	Maintains metabolic homeostasis; promotes mitotic expansion and adipogenesis	Pan et al. (2019), Davies et al. (2015)
CD105 (Endoglin)	Dynamic (low initially → high at 4–7 days)	Acts as a TGF-β co-receptor; high expression inhibits osteogenesis	Sung et al. (2008), Levi et al. (2011)

Abbreviations. ADMSCs, adipose-derived mesenchymal stromal cells; CD, cluster of differentiation; ALP, alkaline phosphatase; TGF, transforming growth factor-β.

This table summarizes representative surface markers of ADMSCs together with their reported functional relevance. Marker expression may vary according to donor characteristics, culture conditions, and passage number.

## 3 MetS-associated pathological state-induced damage to ADMSCs

The MetS-associated cell-microenvironment interaction network is increasingly recognized as a key determinant of ADMSC dysfunction. Rather than acting in isolation, multiple pathological cues collectively reshape ADMSC fate and limit their reparative efficacy. Among these, systemic insulin resistance, chronic inflammation/immune dysregulation, and oxidative stress represent three major and closely interconnected drivers. Collectively, these pathological factors impair ADMSC proliferation, differentiation, immunomodulatory function, and regenerative potential.

Under normal circumstances, adhesion molecules in the Extracellular Matrix (ECM) maintain the homeostasis of MSCs by transducing various mechanical signals (Lim et al., 2022). ECM stiffness serves as a crucial physical factor regulating stromalcells, and the scaffold support and signal guidance it provides constitutes an essential basis for the exertion of normal functions of ADMSCs. However, in the context of MetS, the ECM undergoes significant remodeling, which is mainly characterized by abnormal composition (imbalanced content of collagen, fibronectin, etc.), structural disorganization (fibrosis or scar formation), and imbalanced degradation (dysregulation between matrix metalloproteinases (MMPs) and their inhibitors (TIMPs)) (Hayden et al., 2005; Death et al., 2003). Such remodeling disrupts the homeostasis of the cellular microenvironment and impairs the proliferation, differentiation, and homing capacities of ADMSCs. Among these alterations, aberrant collagen remodeling and the associated changes in ECM mechanics play important regulatory roles in stem cell fate and differentiation potential (Jiang et al., 2024); existing studies have shown that ECM glycoproteins such as laminin are important structural and functional components of the extracellular matrix and contribute to ECM integrity, cell adhesion, and migration (Ishihara et al., 2018). In fibrotic-related microenvironments, ECM-producing fibroblasts are major regulators of matrix remodeling; for example, fibroblast-derived MMP-14 plays a key role in collagen homeostasis and abnormal collagen remodeling may further alter the niche surrounding ADMSCs (Zigrino et al., 2016). Meanwhile, the dysregulated MMP/TIMP balance interferes with the transmission of mechanical signals to MSCs, affecting the migration and proliferation of stem cells (Ries et al., 2007). Owing to the enhanced physical barrier of the ECM, abnormal distribution of adhesion molecules, and disordered chemotactic signals, the homing function of ADMSCs is significantly impaired. The above changes damage the ability of ADMSCs to regulate cell-microenvironment interaction network homeostasis through multiple pathways, exacerbate MetS-associated local cellular disorder and tissue damage, and ultimately form a vicious cycle.

### 3.1 Effects of high-glucose and insulin-resistant conditions on ADMSCs

Insulin resistance (IR), first conceptualized by Reaven as the “insulin resistance syndrome,” represents a core pathological feature of metabolic syndrome (MetS) (Reaven, 1993). In this review, IR is discussed at two related levels. At the systemic level, IR refers to a reduction in systemic insulin sensitivity, which can be assessed using measures such as the oral glucose tolerance test (OGTT) and the insulin tolerance test (ITT). At the cellular level, IR refers to impaired insulin signaling and glucose uptake. When discussing the interaction between insulin resistance and ADMSCs, this review generally refers to the metabolic environment surrounding ADMSCs, such as obesity or type 2 diabetes, together with intracellular signaling abnormalities, particularly in the phosphoinositide 3-kinase/Akt (PI3K/Akt) pathway; when addressing systemic effects, it primarily refers to overall glucose regulation, as indicated by indices such as the Waist-to-Triacylglycerol Index (WTI), triglyceride-glucose index (TyG index), and metabolic score for insulin resistance (METS-IR).

Under physiological conditions, ADMSCs contribute to metabolic homeostasis by supporting adipose tissue remodeling, maintaining insulin sensitivity, and exerting paracrine regulatory effects. Experiments using diabetic rat models further indicate that ADMSCs exposed to a controlled diabetic pathological state can improve glucose metabolism by enhancing insulin sensitivity, promoting pancreatic β-cell regeneration, and attenuating diabetes-related complications (Su et al., 2022).

However, persistent hyperglycemia and systemic insulin-resistant conditions characteristic of MetS disrupt the functional equilibrium between ADMSCs and the metabolic regulatory network. High-glucose stress impairs the metabolic adaptability of ADMSCs, leading to aberrant adipogenic differentiation and reduced activation of insulin signaling pathways. Concurrently, dysfunctional ADMSCs acquire a pro-inflammatory secretory profile and release cytokines that inhibit key insulin signaling mediators, including insulin receptor substrate (IRS), thereby further aggravating the individual’s insulin resistance (Zhou et al., 2025). Compensatory hyperinsulinemia may subsequently develop, thereby exacerbating lipid metabolic dysregulation and promoting MetS progression (Slater, 1991). Consistently, long-term exposure to diets with a high insulin load has been associated with an increased risk of MetS, particularly in women, according to the Hoveyzeh Cohort Study (Elyasi et al., 2024).

As MetS advances toward type 2 diabetes mellitus (T2DM), synergistic disturbances in ADMSC function, epigenetic regulation, and insulin signaling become increasingly evident. In diabetic conditions, epigenetic modifications in insulin resistance-associated genes may shift ADMSCs toward a pro-inflammatory paracrine phenotype (Mirzaeicheshmeh et al., 2021), characterized by enhanced release of pro-inflammatory factors and reduced insulin-sensitizing capacity (Skubis-Sikora et al., 2020). Moreover, hyperglycemia and IR directly impair ADMSC proliferation and migration and are associated with cell-cycle delay/arrest in ADMSCs (Yin et al., 2021), and activate apoptotic pathways involving AURKA-regulated checkpoints and FOXO3a-mediated signaling (Yin et al., 2020), alongside mitochondrial dysfunction and disrupted energy metabolism (Abu-El-Rub et al., 2023). Notably, despite increased senescence and apoptosis, ADMSCs retain core stemness features under diabetic conditions, suggesting potential targets for therapeutic intervention (Cheng et al., 2016).

ADMSCs from diabetic subjects have been reported to exhibit reduced proliferative activity and impaired angiogenic paracrine function, including decreased VEGF secretion (Inoue et al., 2019). Such alterations may weaken endothelial repair capacity and contribute to endothelial dysfunction; endothelial dysfunction, in turn, impairs vasodilation and increases peripheral resistance, thereby favoring hypertension progression (Ma et al., 2023).

The assessment of metabolic indicators and insulin resistance indicators is the conventional approach for diagnosing systemic insulin resistance, which includes the Waist-to-Triacylglycerol Index (WTI), triglyceride glucose index (TyG index), the product of TyG index and abdominal obesity index, and metabolic score of insulin resistance (METS-IR) (Bazyar et al., 2024). Abnormalities in these overall metabolic markers may be closely related to the interactions between ADMSCs and metabolic networks, indirectly reflecting abnormalities in the local adipose tissue microenvironment or in the ADMSCs themselves. For example, elevated triglyceride levels may be associated with abnormal adipogenic differentiation of ADMSCs, leading to fat metabolism disorders. An increase in waist circumference may indicate that functional abnormalities have induced fibrosis of adipose tissue or promoted excessive accumulation of hypertrophic adipocytes.

Collectively, systemic insulin-resistant and hyperglycemic conditions reprogram ADMSCs toward a dysfunctional, pro-inflammatory, and metabolically compromised state, thereby limiting their capacity to restore metabolic and vascular homeostasis.

### 3.2 Effects of inflammatory conditions on ADMSCs

Chronic low-grade inflammation is both a driver and a consequence of metabolic dysfunction in MetS, forming a self-perpetuating cycle between inflammatory signaling and metabolic dysfunction. Pro-inflammatory cytokines such as tumor necrosis factor-α (TNF-α) and interleukin-6 (IL-6) directly impair insulin signaling and reduce cellular insulin sensitivity, while in the local adipose tissue microenvironment, metabolic abnormalities such as elevated circulating free fatty acids and adipose tissue dysfunction further amplify inflammatory responses (Biobaku et al., 2019; Lytrivi et al., 2020). Within this inflammatory–metabolic network, cytokines cooperatively regulate metabolic pathways; for example, TNF-α promotes lipolysis through MEK–ERK activation and increased cAMP signaling (Zhang et al., 2002), and TNF-β has emerged as a potential biomarker linking inflammation to metabolic dysregulation in MetS (Al Asmari et al., 2023).

Under physiological conditions, adipose-derived mesenchymal stromalcells (ADMSCs) play a pivotal role in maintaining inflammatory homeostasis through tightly regulated interactions with immune cells and metabolic signaling pathways. ADMSCs promote macrophage polarization toward the M2 phenotype and suppress pro-inflammatory and pro-fibrotic signaling, including the TGF-β/Smad pathway (Ishiuchi et al., 2023). Additionally, ADMSCs can also release extracellular vesicles (EVs), and these EVs modulate immune responses through multiple mechanisms (Altemus et al., 2022) (Table 3 for details). Collectively, these ADMSC-mediated mechanisms establish an integrated regulatory axis linking stem cells, immune modulation, and metabolic homeostasis.

**TABLE 3:** Representative mechanisms and therapeutic effects of ADMSC-derived exosomes in MetS and related complications.

Disease context	Exosomal cargo/Pathway	Major mechanism	Therapeutic effect	Refs
Immune-metabolic dysregulation	S1P/SK1/S1PR1; PTEN/AKT; AMPK-mediated PPARα/CPT-1A and SREBP-1C/FASN signaling	Immune balance and hepatic lipid metabolism	Anti-inflammatory signaling, immune modulation, metabolic reprogramming	Deng et al. (2019), Liu et al. (2020), Jafarinia et al. (2020), Yang et al. (2023)
Obesity and adipose dysfunction	miR-378; miR-204-5p/Wnt/β-catenin; miR-21/TGF-β	Regulates adipogenic remodeling; reduces inflammation and fibrosis	Improves adipose tissue homeostasis and insulin sensitivity	Ma et al. (2021), Iminitoff et al. (2020), He et al. (2015)
MASLD/MASH	PI3K/AKT/mTOR; CHPT1; miR-150-5p/CXCL1; miR-20a-5p/TGFBR2/p38 MAPK/NF-κB	Anti-fibrotic, anti-inflammatory, EMT inhibition	Suppresses HSC activation and slows fibrosis progression	Zhang et al. (2023), Du et al. (2021), Gan et al. (2023)
Diabetic complications	miR-26a-5p; miR-128-1-5p/TGF-β/Smad axis	Anti-fibrotic, pro-repair, pro-angiogenic	Attenuates renal fibrosis and promotes wound healing/tissue repair	Duan et al. (2020), Liang et al. (2024), Ma et al. (2022)
Endothelial injury/vascular dysfunction	miR-126/PI3K/Akt; miR-342-5p; miR-342-3p/CtBP2/C/EBPα; miR-125a/DLL4/Notch; miR-132/ROCK1/PTEN	Anti-apoptotic, anti-inflammatory, pro-angiogenic	Protects endothelial integrity, reduces atherosclerotic risk, and improves perfusion	Mizuta et al. (2020), Xing et al. (2020), Wang et al. (2015), Liang et al. (2016), Heo and Kim (2022)
Cardiovascular injury/myocardial remodeling	circ-Stt3b/miR-15a-5p/GPX4; miR-671/TGFBR2/Smad2; miR-221/222/p38/NF-κB	Anti-apoptotic, antioxidative, anti-fibrotic	Improves myocardial survival, mitochondrial function, and cardiac repair	Liu et al. (2024b), Wang et al. (2024b), Wang et al. (2021), Lee et al. (2021b)

Abbreviations. ADMSCs, adipose-derived mesenchymal stromal cells; MetS, metabolic syndrome; MASLD, metabolic dysfunction-associated steatotic liver disease; MASH, metabolic dysfunction-associated steatohepatitis; EMT, epithelial–mesenchymal transition; HSCs, hepatic stellate cells; miRNAs, microRNAs.

This table summarizes representative cargos, signaling pathways, mechanisms, and therapeutic effects of ADMSC-derived exosomes in MetS and related complications. The listed items are illustrative rather than exhaustive and are included to highlight the major exosome-mediated mechanisms discussed in the manuscript.

However, obesity-associated inflammatory stress disrupts these beneficial interactions, leading to impaired immunomodulatory function of ADMSCs and further aggravation of metabolic dysfunction. Compared with ADMSCs derived from healthy individuals, those from obese subjects exhibit a markedly reduced capacity to suppress M1 macrophage polarization (Zhu et al., 2021). This dysfunction arises from the synergistic effects of chronic inflammation and high-glucose/high-fat conditions on ADMSC secretory activity. Hyperglycemia downregulates key anti-inflammatory mediators such as indoleamine 2,3-dioxygenase (IDO), IL-10, and complement factor H (Khasawneh et al., 2024), whereas inflammatory stimulation induces excessive secretion of pro-inflammatory cytokines, including IL-6, IL-8, and IL-1β (Buyl et al., 2024), collectively destabilizing immune regulatory balance. Moreover, because adipogenic differentiation and immunomodulatory activity of ADMSCs rely on overlapping metabolic and signaling resources, obesity-associated enhancement of adipogenesis may indirectly weaken the immunoregulatory capacity of ADMSCs (Li et al., 2024). High-fat and high-sugar stimuli further amplify this dysfunction when they act together rather than separately (Xiao et al., 2020).

The relative proliferative stability of ADMSCs does not imply that they are broadly resistant to aging or pathological stress. In the pathological environment of metabolic syndrome, chronic inflammatory stress can offset this relative advantage, accelerate the aging of ADMSCs, and thereby further exacerbate immune dysregulation. Accumulation of oxidative and genotoxic stress in obese adipose tissue activates senescence programs in ADMSCs, resulting in impaired proliferation, altered differentiation, and dysfunctional inflammatory secretory profiles (Galera et al., 2025). Compared with acute inflammatory exposure, prolonged low-grade inflammation induces more persistent damage, ultimately driving ADMSCs into a senescent state with severely compromised capacity to regulate macrophage phenotypic balance and inflammatory homeostasis (Lee HJ. et al., 2021). Collectively, chronic inflammation and immune imbalance reprogram ADMSCs toward a dysfunctional and senescent phenotype, thereby undermining their immunomodulatory capacity and exacerbating metabolic dysfunction in MetS.

### 3.3 Effects of oxidative stress on ADMSCs

Oxidative stress represents a critical pathological mechanism underlying adipose-derived mesenchymal stromalcell (ADMSC) dysfunction in metabolic syndrome. At its source, mitochondrial dysfunction serves as the primary trigger of excessive reactive oxygen species (ROS) generation. Under physiological conditions, mitochondria produce limited amounts of ROS as byproducts of oxidative phosphorylation, which are tightly balanced by intracellular antioxidant systems (Gureev et al., 2023). In metabolically stressed states, mitochondrial structural integrity and respiratory/oxidative phosphorylation (OXPHOS) activity are disrupted, resulting in excessive ROS production and an imbalance between oxidant generation and antioxidant clearance, thereby establishing sustained oxidative stress (Barnett et al., 2025). At this point, the intracellular antioxidant systems (including antioxidant enzymes such as superoxide dismutase SOD, catalase, glutathione peroxidase GPx (Bu et al., 2024), and antioxidants such as vitamin C, vitamin E, and glutathione (Andrés et al., 2024) undergo compensatory activation. However, they can no longer match the rate of excessive ROS accumulation, leading to the establishment of oxidative stress.

ROS directly attack cellular membranes and biomolecules, disrupting redox homeostasis and inducing the accumulation of oxidative products. This damaging effect primarily targets vascular endothelial cells, leading to their dysfunction. Endothelial dysfunction reciprocally impacts the metabolic system through signaling pathways, further inducing typical MetS symptoms such as insulin resistance, hypertension, and atherosclerosis (Small et al., 2018; Vendrov et al., 2024; Wronka et al., 2022). Simultaneously, excessive ROS promotes endothelial activation by increasing adhesion molecule expression, disrupting endothelial junction integrity and barrier function, facilitating inflammatory cell transmigration, and activating inflammatory signaling pathways (Jin et al., 2020). Subsequently, persistent inflammation acts on the metabolic system, further exacerbating metabolic abnormalities.

Excessive ROS directly compromises ADMSC viability and function. Persistent oxidative stress induces mitochondrial network remodeling (Grun et al., 2023), impairs bioenergetic capacity, and weakens intrinsic antioxidant defenses, thereby accelerating cellular senescence and functional decline (Casorati et al., 2025). At the molecular level, ROS disrupt mitochondrial–nuclear communication by interfering with mitochondrial transcription factor A (TFAM) activity and suppressing nuclear enrichment abundance transcript 1 (NEAT1), resulting in impaired DNA repair capacity and activation of senescence- and apoptosis-related pathways (Zhao et al., 2024); These changes collectively reduce ADMSC proliferation, differentiation potential, and migratory capacity (Sela et al., 2015).

Multiple metabolic stressors converge to amplify oxidative damage in ADMSCs. Hyperglycemia destabilizes the mitochondrial electron transport chain, hyperlipidemia promotes lipid peroxidation–mediated ROS accumulation, and chronic inflammation enhances ROS production through NADPH oxidase (NOX)-dependent pathways. The accumulation of ROS, in turn, further activates the NLRP3 inflammasome within the leptin signaling axis, inducing apoptosis in ADMSCs and exacerbating the impairment of their therapeutic efficacy (Nguyen et al., 2025). However, the effects of leptin on ADMSCs are not singularly fixed but exhibit bidirectional regulatory characteristics. *In vitro* and *in vivo* studies have found that leptin may also enhance the therapeutic efficacy of MSCs by targeting the GSK3/OMA1/OPA1 pathway and activating the OPA1/SGLT1 signaling pathway (Yang F. et al., 2019; Yang et al., 2018). Together, these results drive ADMSCs toward a dysfunctional and senescent phenotype, severely limiting their regenerative and immunomodulatory capacities.

Given the central role of oxidative stress in ADMSC impairment, enhancing mitochondrial resilience and ROS tolerance has emerged as a key strategy to restore their therapeutic potential. Genetic interventions, including activation of Akt- or PD-L1–associated pathways, enhance antioxidant signaling and resistance to apoptosis (Lin et al., 2023). Exogenous approaches, such as endothelial cell–derived small extracellular vesicles, deliver bioactive molecules that repair mitochondrial function and improve ADMSC survival under oxidative conditions (Shen et al., 2022). Introducing specific microRNAs from exosomes protects mitochondrial structure and function, helping alleviate oxidative stress and safeguard cellular integrity (Xia et al., 2022). In addition, antioxidant regulators, including NRF1(99) and reduced glutathione (Yu et al., 2025), promote mitochondrial biogenesis and ROS scavenging. Notably, photobiomodulation represents a non-invasive strategy that restores redox balance and significantly improves human-ADMSC proliferation, migration, and differentiation, highlighting its translational potential (Pan et al., 2022).

## 4 ADMSCs-based therapies for metabolic syndrome

### 4.1 Therapeutic potential of ADMSCs for obesity

Dysregulated lipid metabolism is a core pathological feature of obesity and MetS, leading to elevated circulating free fatty acid (FFA) flux, which in turn contributes to the development and exacerbation of metabolic diseases (Grabner et al., 2021). Concurrently, persistent mild inflammatory responses, oxidative stress imbalances, and abnormal adipose tissue remodeling (such as the coexistence of pathological fat accumulation and functional adipose tissue deficiency) intertwine to form a vicious cycle that drives disease progression. Adipose tissue serves as both a natural niche and a primary target for MSC therapy. Accordingly, the therapeutic role of ADMSCs in obesity can be understood through three biologically relevant modes of interaction: autocrine regulation within ADMSCs, paracrine interactions within the adipose niche, and endocrine interactions with distal tissues.

At the level of autocrine regulation, obesity-related signals reshape the intrinsic regulatory state of ADMSCs and thereby influence their adipogenic and metabolic functions. Peroxisome proliferator-activated receptor gamma (PPARγ) is a central regulator of adipocyte differentiation and lipid metabolism; its transcriptional activity is altered under pathological conditions associated with obesity. Current evidence suggests that, PPARγ transcriptional activity in ADMSCs can be modulated by upstream inflammatory and adipogenic regulatory signals, including TNF-α-, IL-1, and TGF-β-related signaling, as well as by adipogenesis-associated regulators such as FABP4 (Garin-Shkolnik et al., 2014; Takada et al., 2010). Specifically, cytokine-related signaling and FABP4-related regulation may alter PPARγ activity in ADMSCs, thereby influencing adipogenic differentiation and lipid-handling programs. At the functional level, modulation of this pathway may contribute to improved adipose tissue remodeling by supporting more appropriate adipocyte differentiation and lipid storage, thereby limiting ectopic fat accumulation. Consistent with this possibility, ADMSC-based interventions have been associated with favorable changes in lipid metabolism-related gene expression and circulating lipid parameters, including reduced triglyceride and LDL-C levels and increased HDL-C, which may further alleviate vascular lipid deposition and cardiovascular risk (Cao et al., 2015).

Notably, this autocrine regulatory axis is closely linked to insulin signaling. After ADMSCs upregulated the expression of IRS-1 (Mohammed et al., 2024), the activated IRS-1/PI3K/Akt signaling pathway could reverse enhance the functional activation of PPARγ, and the moderate activation of PPARγ could also enhance insulin signaling efficiency by promoting IRS-1 phosphorylation, forming a positive cycle of lipid metabolism regulation and insulin sensitization, jointly alleviating metabolic disorders of fat cells. In addition, ADMSCs can simultaneously regulate glucose metabolism and energy expenditure, further optimizing the synergistic imbalance of glycolipid metabolism in the pathological state of obesity (Ma et al., 2021). Recent animal studies have shown that ADMSCs may regulate the expression of adipogenesis-related genes and ameliorate metabolic disorders in pigs with MetS and renovascular hypertension by reducing inflammatory responses (Krueger et al., 2025). Sustained suppression of inflammation has been proposed as an effective strategy for preventing obesity-related metabolic dysfunction (Wang et al., 2022).

At the level of paracrine interactions within the adipose niche, the therapeutic role of ADMSCs depends more directly on their communication with neighboring adipocytes, resident macrophages, stromal cells, and local inflammatory or fibrotic cues. In dysfunctional adipose tissue, these interactions shape local tissue homeostasis and determine whether ADMSCs can effectively regulate inflammation, adipogenic remodeling, fibrosis, and metabolic balance (Zhou et al., 2023; Tao et al., 2025). Through the secretion of cytokines, extracellular vesicles, and exosome-associated microRNAs, adipose-derived mesenchymal stromalcells may regulate local inflammation, adipogenic remodeling, fibrosis, and metabolic balance. In particular, ADMSCs-secreted exosomes not only exert direct anti-inflammatory and antioxidant effects (Liu et al., 2022), but more importantly, the miRNAs carried within these exosomes can directly regulate adipogenic differentiation in both donor and target cells. In the pathological state of obesity, these exosome-mediated regulatory networks may intersect with inflammatory pathways, insulin-related signaling pathways, and adipogenesis-related processes, thereby contributing to the improvement of adipose tissue remodeling and the restoration of microenvironmental homeostasis. These exosome-mediated mechanisms are summarized in Table 3.

Based on the above paracrine features, targeted intervention strategies can achieve precise alleviation of the pathological microenvironment of local adipose tissue by regulating key pathways. In pathological scenarios of insufficient functional adipose tissue, modulation of miR-378-related pathways has been proposed as a potential means to promote adipogenic remodeling and metabolic adaptation (Li et al., 2021). Simultaneously, through its interactions with the PPARγ and IRS-1/PI3K/Akt pathways, it promotes the formation of healthy adipose tissue while concurrently improving insulin sensitivity. In the pathological state of ectopic fat accumulation, miR-34a inhibition can prevent excessive lipid differentiation and pathological fat deposition (Park et al., 2015). Together, these findings support the view that paracrine interactions within the adipose niche represent a central mechanism through which ADMSCs exert local therapeutic effects in obesity.

Beyond the adipose niche, these local effects extend to endocrine interactions with distal tissues. As noted above, improved adipose tissue remodeling may reduce the release of circulating free fatty acids, mitigate ectopic lipid deposition, and promote systemic metabolic homeostasis. These distal metabolic effects are particularly relevant in obesity, as adipose dysfunction is closely linked to other metabolically active organs. In this regard, the adipose-liver axis is of particular importance, as excessive lipid flux, inflammatory mediators, and systemic insulin resistance resulting from adipose tissue dysfunction directly contribute to hepatic steatosis and metabolic damage (Azzu et al., 2020). Thus, the beneficial effects of ADMSCs in obesity should not be viewed as confined to adipose tissue alone, but rather as part of a broader interorgan regulatory network linking local niche remodeling to systemic metabolic outcomes.

Taken together, ADMSC-mediated regulation in obesity is best understood across three interconnected levels—autocrine regulation, paracrine interactions within the adipose niche, and endocrine interactions with distal tissues—which together link local adipose remodeling to broader systemic metabolic outcomes. Given the biological and functional link between liver disease and lipid metabolism disorders, the following section will discuss the role of ADMSCs in liver disease.

### 4.2 Therapeutic potential of ADMSCs for liver disease (MASLD/MASH)

Metabolic dysfunction-associated steatotic liver disease (MASLD; formerly NAFLD), the hepatic manifestation of MetS, may progress from simple steatosis to metabolic dysfunction-associated steatohepatitis (MASH; formerly NASH) and fibrosis (Han L. et al., 2025), driven by lipotoxicity, mitochondrial dysfunction, IR, inflammation, and oxidative stress (Cai et al., 2025). Current therapies, including lifestyle modification and pharmacological interventions, may partially alleviate symptoms in the early stages; nevertheless, they are generally ineffective at reversing fibrosis and may be associated with adverse effects, highlighting the clinical necessity for the exploration of stem cell-based therapies (Sinn et al., 2023). Furthermore, ADMSCs show superior efficacy in promoting the healing and regenerative capacity of liver tissues and ameliorating hepatic fibrosis compared with other types of MSCs in mouse models of liver injury (Elzainy et al., 2024).

Although this section primarily examines the effects of ADMSCs on the liver microenvironment, it is important to note that these local changes can produce systemic metabolic effects by modulating hepatic metabolic pathways, thereby alleviating MASLD and linking local actions to systemic glucose and lipid homeostasis. ADMSCs exert therapeutic effects in MASLD through multiple mechanisms, including mitochondrial protection, insulin sensitization, anti-inflammatory signaling, and anti-fibrotic regulation. Because the first three mechanisms have been described in detail in the previous sections, this section primarily emphasizes the anti-fibrotic effects of ADMSCs in the hepatic microenvironment.

The core therapeutic advantage of ADMSCs lies in their ability to target the fibrotic state driven by activated hepatic stellate cells (HSCs). As the “effector cells” of liver fibrosis, HSCs are persistently activated to a myofibroblastic phenotype within the MASLD pathological microenvironment. They secrete large amounts of extracellular matrix components such as collagen and actively promote the fibrotic process. Hepatocyte growth factor (HGF) secreted by ADMSCs directly inhibits hepatic stellate cell activation and promotes collagen degradation in fibrotic liver tissue, thereby attenuating fibrosis (Yu et al., 2023). This process exhibits core synergistic regulation with the YAP/TAZ signaling axis. Abnormal activation of the YAP/TAZ signaling axis in MASLD pathology serves as a key pathway driving HSC activation. ADMSCs can precisely downregulate this signaling axis to inhibit HSC phenotypic conversion (Liu H. et al., 2024), while HGF secretion further enhances the inhibitory effect on YAP/TAZ signaling.

Beyond the direct anti-fibrotic actions of ADMSCs, ADMSC-derived exosomes provide an additional cell-free mechanism for regulating the hepatic fibrotic microenvironment. Available studies suggest that these exosomes can inhibit hepatic stellate cell activation, attenuate pro-fibrotic signaling, and modulate inflammatory and metabolic pathways involved in liver fibrosis progression. The representative exosomal cargos and signaling mechanisms are summarized in Table 3.

Given the complexity of the MASLD-associated pathological microenvironment, the therapeutic efficacy of ADMSCs may be further enhanced through targeted optimization. Under the influence of the MetS-associated cell-microenvironment interaction network, ADMSCs may exhibit insufficient endogenous secretory activity. Exogenous supplementation of HGF can synergistically enhance the anti-inflammatory effects and collagen deposition reduction of ADMSCs (Gharbia et al., 2022). Pre-treatment with Eugenol enhances the homing capacity of rat-ADMSCs, enabling them to more precisely target inflammatory-fibrotic lesions in the liver, thereby more effectively improving liver function and reducing serum fibrosis markers (Fathy et al., 2020). Ferulic acid pretreatment targets early-stage fatty degeneration in MASLD by enhancing ADMSCs’ ability from obese mice to regulate lipid metabolism, thereby reducing the damage caused by steatohepatotoxicity to the hepatic microenvironment (Cho and Park, 2020). Optimizing relevant variables to enhance the adaptability of ADMSCs to the MASLD pathological microenvironment and provide a more effective way for the treatment of MASLD.

Although MASLD/MASH is the primary hepatic manifestation of MetS, progressive metabolic dysfunction is not limited to the liver. Persistent insulin resistance, chronic inflammation, and disturbances in glucose and lipid homeostasis further exacerbate the deterioration of endocrine and metabolic function, ultimately leading to diabetes, another key disease state resulting from the progression of MetS.

### 4.3 Therapeutic potential of ADMSCs for diabetes

Against a backdrop of reduced insulin sensitivity and impaired hepatic glucose metabolism, ADMSCs have been reported to improve insulin sensitivity partly by modulating hepatic glucose metabolism under pathological conditions (Xie et al., 2017; Wang M. et al., 2018). Notably, *in vitro* studies have shown that human ADMSCs can be induced to differentiate into insulin-producing cells (IPCs) that secrete insulin within a hyperglycemic pathological state, thereby contributing to insulin replacement and improving glycemic control (Wang R. et al., 2018) (Moshtagh et al., 2013). This strategy is particularly relevant to the pathological state of T1DM, in which immune-mediated destruction of IPCs within the islets constitutes the core pathological feature. Regenerative therapy using ADMSC-derived IPCs may help restore islet function through cellular replacement, although immune and autoimmune injury remain important challenges for translation (Shi et al., 2026). Furthermore, compared to induced pluripotent stem cells (iPSC)-derived IPCs, ADMSC-derived IPCs remain at an earlier stage of translational development. Pluripotent stem cell-derived products have shown more advanced functional maturation and early clinical proof-of-concept in human recipients (Balboa et al., 2022; Wang S. et al., 2024), whereas evidence for ADMSC-derived IPCs is still mainly limited to *in vitro* induction and diabetic animal models (Wada et al., 2019). Nevertheless, ADMSC-derived IPC strategies may retain practical advantages such as relatively accessible cell procurement and potential autologous use (Ikemoto et al., 2019).

Beyond cell replacement, ADMSCs also exert potent paracrine effects that target insulin signaling pathways compromised by chronic inflammation and hyperglycemia. Through the secretion of cytokines and microRNAs, ADMSCs directly modulate key components of the insulin signaling cascade under pathological conditions (Conle et al., 2018). ADMSC-derived paracrine factors have been reported to improve insulin signaling–related pathways under pathological conditions; in particular, ADMSC-derived fibroblast growth factor-1 (FGF-1) has been implicated in the upregulation of insulin receptor substrate-1 (IRS-1) and glucose transporter 4 (GLUT4) in an *in vitro* insulin-resistance model (Kim et al., 2018). By enhancing IRS-1 phosphorylation and downstream PI3K/Akt activation, FGF-1 promotes GLUT4 translocation to the plasma membrane and thereby improves glucose uptake and insulin sensitivity (Wang et al., 2019).

Beyond improving insulin sensitivity and suppressing chronic inflammation, ADMSCs may also ameliorate diabetes-related complications. In addition to direct cellular and paracrine effects, ADMSC-derived extracellular vesicles carry bioactive RNAs and signaling molecules that contribute to tissue repair, angiogenesis, and anti-fibrotic regulation in pathological hyperglycemic settings. Accordingly, exosome-mediated signaling may complement the glucose-regulating and insulin-sensitizing effects of ADMSCs in diabetic nephropathy and delayed wound healing. These exosome-mediated mechanisms are summarized in Table 3.

To address the issue of suboptimal cell therapy efficacy caused by the hyperglycemic pathological state, combined intervention strategies demonstrate optimization potential. Betatrophin, as a factor promoting pancreatic beta cell proliferation, enhances the insulin secretion function induced by ADMSCs when co-cultured with them, offering a new direction for replacing insulin injections (Sun et al., 2017). To address potential immune rejection and apoptosis-related issues in cell therapy, *in vitro* studies have demonstrated that co-administration of tacrolimus (FK506) may reduce cytotoxicity by suppressing immune responses, decreasing apoptosis in ADMSCs, and further enhancing therapeutic efficacy (Sir et al., 2018). Additionally, metformin-pretreated ADMSCs demonstrated synergistic effects in a high-fat mouse model, improving multiple metabolic abnormalities within the complex pathological state of MetS, which is characterized by the coexistence of hyperglycemia, hyperinsulinemia, and hypertriglyceridemia (Shree and Bhonde, 2016). However, particular attention should be paid to the potential adverse effect of metformin-induced stem cell apoptosis under intensive glucose control, as this may diminish stem cell-mediated therapeutic efficacy in diabetic settings and warrants caution in combination therapy (He et al., 2019).

As metabolic dysfunction progresses, the impact of persistent hyperglycemia, insulin resistance, and chronic inflammation on vascular homeostasis becomes increasingly pronounced. Consequently, endothelial damage represents a major downstream consequence of diabetes and metabolic syndrome. Based on this, we will focus on exploring the therapeutic potential of ADMSCs in vascular protection, particularly in the context of endothelial dysfunction and vascular inflammatory damage.

### 4.4 Therapeutic potential of ADMSCs for vascular protection

Metabolic syndrome is often accompanied by a complex pathologic microenvironment of various types of local vascular endothelial injury: metabolic abnormalities such as hyperglycemia and hyperlipidemia directly induce oxidative stress imbalance in endothelial cells. Simultaneously, they activate inflammatory signaling pathways, promoting the adhesion and migration of inflammatory cells to the vascular wall, thereby exacerbating endothelial inflammatory responses.

ADMSCs improve endothelial function, reduce inflammatory cell adhesion, and enhance tissue perfusion within the pathological microenvironment of endothelial injury in MetS through an exosome-mediated miRNA regulatory network. This characteristic confers a potential therapeutic advantage for alleviating MetS (141). By delivering regulatory microRNAs and other signaling cargos, they help reduce endothelial apoptosis, suppress inflammatory activation, and promote angiogenesis, thereby improving endothelial integrity and tissue perfusion in Mets. The detailed signaling pathways and representative cargos are discussed in Table 3.

Several clinical trials are currently underway aimed at exploring the efficacy of ADMSCs in protecting the vascular endothelium. A cohort study on autologous ADMSC therapy for atherosclerosis patients fully considered the individual variability in the pathological state of endothelial injury among MetS patients, employing autologous cell therapy to enhance biocompatibility. Results demonstrated that not only were no significant adverse reactions observed post-treatment, but improvements in atherosclerotic conditions were also achieved, providing direct evidence for clinical translation (Ohta et al., 2020). Additionally, the obesity-associated hypoxic environment can modulate the expression of angiogenesis-related genes in mice to some extent and significantly alter the phenotype of ADMSCs (Kang et al., 2023), particularly by changing the expression patterns of cell surface markers on ADMSCs, thereby affecting therapeutic efficacy (Pachón-Peña et al., 2016).

These findings highlight the importance of local endothelial microenvironmental interactions in vascular protection. Building on this, we will now discuss the broader implications of ADMSCs for systemic cardiovascular disease.

### 4.5 Therapeutic potential of ADMSCs for cardiovascular disease

The previous section discussed how ADMSCs exert a protective effect on vascular cells through interactions with the local microenvironment, such as by secreting growth factors and anti-inflammatory cytokines to promote local vascular repair and protect damaged blood vessels from ischemic injury. These local effects directly improve endothelial function and reduce local inflammation, thereby enhancing vascular protection. At the same time, ADMSCs also exert systemic effects. Factors released into the circulatory system can modulate immune responses in distant tissues and reduce systemic inflammation, thereby further improving overall cardiovascular function. Consequently, the local vascular protection mediated by ADMSCs in the microenvironment ultimately translates into broader systemic cardiovascular benefits.

Atherosclerosis and myocardial injury constitute the core pathological basis of cardiovascular disease (CVD). While conventional therapies alleviate symptoms, they fail to target the repair of damaged myocardial tissue or reverse the vicious cycle of cardiovascular disease. ADMSCs, with their precise adaptability to the cardiovascular pathological conditions, demonstrate therapeutic advantages over BM-MSCs, positioning them as more promising therapeutic candidates (Kakkar et al., 2019).

In response to energy metabolism depletion and uncontrolled myocardial cell apoptosis induced by the progression of cardiovascular disease, ADMSCs achieve preliminary restoration of myocardial contractile function through the close synergy of energy metabolism repair and anti-apoptotic mechanisms. Under myocardial ischemia and hypoxia, mitochondrial dysfunction impairs ATP synthesis, leading to reduced myocardial contractility. ADMSCs can upregulate mitochondrial transporter expression in myocardial tissue, enhance mitochondrial oxidative phosphorylation efficiency, and significantly increase ATP concentration, thereby improving myocardial energy metabolism and contractile function (Mori et al., 2021). This energy-restoration process acts synergistically with anti-apoptotic mechanisms: exosomes secreted by ADMSCs suppress the expression of apoptosis-related proteins, including Bax, caspase-3, and p53 (Wang et al., 2023), thereby reducing cardiomyocyte apoptosis. The preservation of viable myocardial cells also provides a cellular basis for the upregulation of mitochondrial transporters, thereby establishing a positive feedback loop.

Oxidative stress is a key contributor to myocardial ischemia-reperfusion injury and subsequent fibrosis. ADMSC-EVs can suppress oxidative stress through multiple pathways; the detailed cargos and signaling pathways involved are summarized in Table 3. Alleviating oxidative stress simultaneously creates a favorable microenvironment for anti-angiogenic mechanisms. Angiogenic factors such as VEGF secreted by ADMSCs can enhance the tube-forming capacity of endothelial cells (Nauta et al., 2013), improve myocardial microcirculation, alleviate ischemia and hypoxia, and further inhibit oxidative stress and apoptosis. Notably, hypoxic pretreatment can mimic the microenvironment of localized myocardial ischemia, activate the adaptive stress response of ADMSCs, and significantly enhance the biological activity of ADMSC-EVs (Mao et al., 2021), thereby optimizing therapeutic outcomes.

Under the pathology of cardiomyocyte loss and functional reconstruction, the targeted differentiation mechanism of ADMSCs synergizes with microenvironmental regulatory factors to ensure precise repair of cardiac function. Patients with arrhythmia exhibit pathological characteristics of pacemaker cell dysfunction. By leveraging SK4 gene overexpression technology, mice-ADMSCs can be directed to differentiate into pacemaker-like cells, directly compensating for cellular functional deficits in pathological states (Yang M. et al., 2019). For massive myocardial cell necrosis caused by conditions like myocardial infarction, on specific scaffolds (Tambrchi et al., 2022), inducers such as ghrelin can activate the DDX17/SFRP4/Wnt/β-catenin signaling axis to synergistically trigger the differentiation of ADMSCs into functional cardiomyocytes (Liu et al., 2023).

Through coordinated effects on myocardial energetics, oxidative stress, angiogenesis, and targeted differentiation, ADMSCs offer a multifaceted strategy for functional cardiac repair in cardiovascular disease.

### 4.6 ADMSC-derived exosomes as a cell-free therapeutic strategy

ADMSC-derived exosomes have emerged as a promising cell-free therapeutic modality in MetS. As nanoscale extracellular vesicles enriched with bioactive cargos, including microRNAs, proteins, lipids, and signaling molecules, exosomes can recapitulate many of the paracrine benefits of parental ADMSC (Liu et al., 2021). The diverse molecular constituents of exosomes endow them with distinct biological functions. In particular, their protein cargos, including membrane receptors, signaling proteins, enzymes, and other membrane-associated proteins, play critical roles in intercellular signaling, targeting and uptake, membrane fusion-related events, and molecular cargo transfer (Kumar et al., 2024). By delivering bioactive cargo, including cytokine-related proteins, receptors, signaling proteins, and regulatory RNAs, exosomes can activate or suppress intracellular signaling pathways, thereby regulating cellular functions and showing therapeutic promise in diseases characterized by chronic inflammation, oxidative stress, metabolic dysregulation, and multi-organ injury (Isaac et al., 2021).

Mechanistically, ADMSC-derived exosomes exert broad regulatory effects across MetS-related pathologies through coordinated anti-inflammatory, antioxidative, pro-angiogenic, anti-fibrotic, and metabolic reprogramming pathways (Liu et al., 2018; Li et al., 2018; Zhang et al., 2023). In diverse experimental and disease settings, ADMSC-derived exosomes have demonstrated significant therapeutic potential for the treatment of MetS-associated disorders, as summarized in Table 3.

## 5 Risks, challenges, and potential optimization strategies of treatment

### 5.1 Risks and challenges

Although preclinical studies have demonstrated the promising therapeutic potential of ADMSCs in MetS, further well-designed clinical studies are required before these findings can be translated into clinical practice. Given the complex pathophysiology of MetS, the safety and efficacy of ADMSC-based therapy require validation in larger and more comprehensive clinical trials. Because the long-term adverse effects and durability of efficacy of ADMSC therapy remain long-term follow-up strategies are essential for appropriate evaluation.

The clinical use of MSCs may be associated with tumor-related risks, and evidence suggests that these cells may be involved in the progression of several malignancies, including ovarian, breast, and colorectal cancers (McLean et al., 2011; Chaturvedi et al., 2013; Zhang et al., 2018). However, this association has not been definitively established, and conflicting findings have been reported in preclinical and experimental studies. For example, in breast cancer models, ADMSCs have been reported to promote tumor growth or metastatic potential through multiple mechanisms (Ritter et al., 2023), whereas other preclinical studies have shown that ADMSC-derived exosomes may exert potential anti-tumor effects by inhibiting cancer cell proliferation and regulating the tumor microenvironment (Li et al., 2020). Although the mechanisms underlying the bidirectional interaction between MSCs and cancer remain incompletely elucidated, these conflicting findings highlight the complexity of the role of ADMSCs in tumor biology; their effects may depend on factors such as tumor type, the phenotypic characteristics of ADMSCs, and the local microenvironment. This duality underscores the potential risks of ADMSC therapy and highlights the need for a more comprehensive understanding of cancer-related mechanisms before clinical application.

At present, standardized methods are needed for isolating, processing, and preserving ADMSCs, potentially leading to inconsistent quality of cell products and adversely impacting treatment and patient safety. In addition, the economic burden of stem cell therapy is considerably elevated, encompassing the processes of collecting, cell culture, and transplantation, which may limit its popularization and application in clinical practice.

These limitations have driven increasing interest in the development of alternative and optimized therapeutic approaches.

### 5.2 Potential optimization strategies

Table 4 summarizes several key targets of ADMSC-based therapy for MetS as described above. In addition, one promising approach is the use of ADMSC-derived exosomes as a cell-free therapeutic alternative, which may reduce several risks associated with direct cell transplantation while offering advantages in storage and delivery. Exosomes carry diverse bioactive cargos, and as a cell-free therapeutic platform, generally exhibit lower immunogenicity, reduced infusion-related toxicity, and favorable biocompatibility during *in vivo* delivery (Tan et al., 2024). Exosomes represent a cell-free alternative to stem cell therapy, potentially avoiding several limitations associated with direct cell transplantation (Lou et al., 2017), while also offering practical advantages in preservation and storage.

**TABLE 4:** Key signaling pathways and therapeutic effects of ADMSCs in MetS.

Pathological context	Representative signaling pathways/Targets	Main ADMSC function	Therapeutic effects	Representative refs
Insulin resistance/glucose dysregulation	Hepatic glucose metabolism-related pathways; differentiation into IPCs; FGF-1/IRS-1/PI3K/Akt/GLUT4 axis	Improve insulin sensitivity; enhance glucose uptake and utilization; compensate for impaired insulin secretion	Alleviates insulin resistance, improves glucose homeostasis, and supports glycemic control	Xie et al. (2017), Wang et al. (2018a), Wang et al. (2018b), Moshtagh et al. (2013), Shi et al. (2026), Conle et al. (2018), Kim et al. (2018), Wang et al. (2019)
Diabetes-associated complications	miR-26a-5p; miR-128-1-5p/TGF-β/Smad axis; endothelial repair-related signaling	Promote paracrine repair; anti-fibrotic regulation; pro-angiogenic tissue repair	Attenuates diabetic nephropathy-related fibrosis and promotes wound healing/tissue repair	Duan et al. (2020), Liang et al. (2024), Ma et al. (2022)
Chronic inflammation/immune imbalance	Macrophage polarization; TGF-β/Smad; S1P/SK1/S1PR1; PTEN/AKT; AMPK-mediated PPARα/CPT-1A and SREBP-1C/FASN signaling	Immunomodulation; suppression of chronic low-grade inflammation; restoration of immune-metabolic balance	Reduces inflammatory injury, enhances tissue repair, and metabolic homeostasis	Ishiuchi et al. (2023), Altemus et al. (2022), Deng et al. (2019), Liu et al. (2020), Jafarinia et al. (2020), Yang et al. (2023)
Oxidative stress/mitochondrial dysfunction	TFAM/NEAT1-associated mitochondrial-nuclear communication; NLRP3 inflammasome; Akt- and PD-L1-associated protective signaling; NRF1; antioxidant regulation	Restore redox homeostasis; preserve mitochondrial integrity; improve stress resistance	Attenuates oxidative damage, improves mitochondrial function, delays senescence, and preserves regenerative capacity	Grun et al. (2023), Casorati et al. (2025), Zhao et al. (2024), Sela et al. (2015), Nguyen et al. (2025), Yang et al. (2019a), Yang et al. (2018), Lin et al. (2023), Shen et al. (2022), Xia et al. (2022), Lee et al. (2025), Yu et al. (2025), Pan et al. (2022)
Obesity/adipose tissue dysfunction	Adipogenesis-related signaling; miR-378; miR-204-5p/Wnt/β-catenin; miR-21/TGF-β; PPARγ and IRS-1/PI3K/Akt pathways	Regulate adipogenesis and lipid metabolism; reduce inflammation and fibrosis; improve adipose tissue homeostasis	Improves insulin sensitivity, limits pathological fat accumulation, and alleviates obesity-associated metabolic disturbance	Ma et al. (2021), Krueger et al. (2025), Wang et al. (2022), Liu et al. (2022), Li et al. (2021), Park et al. (2015), Iminitoff et al. (2020), He et al. (2015)
MASLD/MASH	HGF; YAP/TAZ; PI3K/AKT/mTOR; CHPT1; miR-150-5p/CXCL1; miR-20a-5p/TGFBR2/p38 MAPK/NF-κB	Inhibit hepatic stellate cell activation; suppress fibrogenic signaling; regulate inflammation-fibrosis	Ameliorates liver fibrosis, improves liver repair, and alleviates steatotic liver injury	Yu et al. (2023), Liu et al. (2024a), Zhang et al. (2023), Du et al. (2021), Gan et al. (2023)
Endothelial injury/vascular dysfunction	miR-126/PI3K/Akt; miR-342-5p; miR-342-3p/CtBP2/C/EBPα; miR-125a/DLL4/Notch; miR-132/ROCK1/PTEN	Protect vascular endothelium; inhibit apoptosis and inflammatory adhesion; promote angiogenesis	Improves endothelial integrity, enhances tissue perfusion, and lowers vascular injury/atherosclerotic progression	Immanuel and Yun (2023), Ohta et al. (2020), Mizuta et al. (2020), Xing et al. (2020), Wang et al. (2015), Liang et al. (2016), Heo and Kim (2022)
Cardiovascular injury/myocardial remodeling	Mitochondrial oxidative phosphorylation support; Bax/caspase-3/p53 suppression; circ-Stt3b/miR-15a-5p/GPX4; miR-671; miR-221/222/p38/NF-κB; VEGF-related angiogenesis	Improve myocardial energy metabolism; inhibit apoptosis, oxidative stress, and fibrosis; support microvascular repair	Enhances myocardial survival and contractile function, reduces ischemic/oxidative injury, and promotes cardiac repair	Mori et al. (2021), Wang et al. (2023), Nauta et al. (2013), Mao et al. (2021), Liu et al. (2024b), Wang et al. (2024b), Wang et al. (2021), Lee et al. (2021b)

Abbreviations. ADMSCs, adipose-derived mesenchymal stromal cells; MetS, metabolic syndrome; IPCs, insulin-producing cells; HGF, hepatocyte growth factor; NRF1, nuclear respiratory factor 1; TFAM, mitochondrial transcription factor A; MASLD, metabolic dysfunction-associated steatotic liver disease; MASH, metabolic dysfunction-associated steatohepatitis.

This table summarizes the representative signaling pathways, principal biological functions, and therapeutic effects of ADMSCs in MetS and its related complications. The listed pathways are intended to highlight major mechanistic axes discussed in the manuscript rather than provide an exhaustive catalog.

Additionally, as a heterogeneous syndrome, MetS presents substantial challenges for stem cell therapy due to the diversity of its complications—such as type 2 diabetes, obesity, and metabolic dysfunction-associated steatotic liver disease—as well as marked interpatient pathological heterogeneity. Therefore, ADMSC therapy should be tailored to the dominant clinical phenotype of each patient. For patients primarily presenting with hyperglycemia, therapeutic strategies should focus on alleviating insulin resistance and repairing pancreatic β-cells to enhance glucose-lowering cellular phenotypes. For those with liver fibrosis, priority should be given to suppressing hepatic stellate cell activation. For those with adipose inflammation, anti-inflammatory and adipogenesis-regulating functions should be preferentially enhanced.

To further overcome the suppression of ADMSCs’ function by the MetS-associated cell-microenvironment interaction network, exogenous intervention is indispensable: gene editing technologies can customize cellular functions. Targeting NRIP1, a transcription co-repressor that inhibits thermogenesis in ADMSCs, CRISPR/Cas9-mediated knockout of this gene, followed by transplantation into high-fat diet-induced obese mice, reduced adipose and hepatic lipid accumulation while improving glucose tolerance (Tsagkaraki et al., 2021). Additionally, the CRISPR-Cas9-SAM-gRNA enhancement technique can be employed to activate the expression of the endogenous thermogenic regulator UCP1 within ADMSCs, inducing their differentiation into human brown-like (HUMBLE) cells (Wang CH. et al., 2020). This process subsequently improves glucose homeostasis and insulin sensitivity in obese mice. Previous studies have demonstrated that genetically modified engineered MSCs can stably release GLP-1, effectively improving glucose tolerance and alleviating insulin resistance in diabetic mice (Huang et al., 2026). Currently, this strategy faces challenges, including assessing the functional heterogeneity of autologous ADMSCs and validating the long-term safety of exogenous interventions.

In view of the above challenges, the utilization of ADMSCs for the treatment of MetS remains a protracted endeavor. It is imperative to validate the efficacy and safety of ADMSCs in the treatment of MetS and to develop and implement a uniform and standardized technique for the extraction, preparation, and storage. In addition, strategies to decrease the expenses associated with stem cell therapy should be investigated to enhance its applicability across various clinical environments. These efforts are expected to transform ADMSC therapy into a dependable alternative for the management of MetS, offering renewed therapeutic optimism for patients.

## 6 Conclusion

This review provides a comprehensive overview of the potential application of ADMSCs in the treatment of MetS, highlighting their multifaceted therapeutic mechanisms and overall therapeutic promise. Current evidence suggests that ADMSCs have significant effects on alleviating systemic insulin resistance, regulating inflammatory response, repairing mitochondrial damage and oxidative stress, reducing obesity, ameliorating MASLD, and cardiovascular disease. However, the cell-microenvironment interaction network of MetS itself can impair ADMSC function. Factors such as chronic inflammation, heightened oxidative stress, and metabolic abnormalities may lead to reduced proliferative activity, diminished differentiation capacity, and decreased secretion of therapeutic cytokines by ADMSCs. These findings provide a critical perspective for deepening our understanding of the interaction between ADMSCs and MetS.

In parallel, ADMSC–derived exosomes have emerged as a promising cell-free therapeutic alternative, offering immunomodulatory and regenerative benefits while potentially reducing some risks associated with cell-based therapies. Nonetheless, rigorous evaluation of their safety, efficacy, and translational feasibility remains essential.

Overall, ADMSC–based approaches represent an innovative and versatile strategy for addressing MetS. Future studies should focus on elucidating cell-microenvironment interaction-driven mechanisms that constrain ADMSC function, systematically assessing the safety and therapeutic efficacy of ADMSCs and their derivatives, and developing optimization strategies to enhance resistance to pathological stress, such as CRISPR-based genetic modification, exogenous preconditioning, and engineered MSC platforms. These efforts will advance the clinical translation of ADMSC-based therapies and open new avenues for personalized and precision medicine in metabolic diseases.
